# Neuro-oncological augmented reality planning for intracranial tumor resection

**DOI:** 10.3389/fneur.2023.1104571

**Published:** 2023-03-14

**Authors:** Frederick Van Gestel, Taylor Frantz, Felix Buyck, Wietse Geens, Quentin Neuville, Michaël Bruneau, Bart Jansen, Thierry Scheerlinck, Jef Vandemeulebroucke, Johnny Duerinck

**Affiliations:** ^1^Department of Neurosurgery, Universitair Ziekenhuis Brussel (UZ Brussel), Vrije Universiteit Brussel (VUB), Brussels, Belgium; ^2^Research Group Center for Neurosciences (C4N-NEUR), Vrije Universiteit Brussel (VUB), Brussels, Belgium; ^3^Department of Electronics and Informatics (ETRO), Vrije Universiteit Brussel (VUB), Brussels, Belgium; ^4^IMEC, Leuven, Belgium; ^5^Department of Orthopedic Surgery and Traumatology, Universitair Ziekenhuis Brussel (UZ Brussel), Vrije Universiteit Brussel (VUB), Brussels, Belgium; ^6^Research Group Beeldvorming en Fysische Wetenschappen (BEFY-ORTHO), Vrije Universiteit Brussel (VUB), Brussels, Belgium; ^7^Department of Radiology, Universitair Ziekenhuis Brussel (UZ Brussel), Vrije Universiteit Brussel (VUB), Brussels, Belgium

**Keywords:** augmented reality, intracranial tumor, resection planning, neuronavigation, brain tumor, computer-assisted surgery, preoperative preparation

## Abstract

**Background:**

Before starting surgery for the resection of an intracranial tumor, its outlines are typically marked on the skin of the patient. This allows for the planning of the optimal skin incision, craniotomy, and angle of approach. Conventionally, the surgeon determines tumor borders using neuronavigation with a tracked pointer. However, interpretation errors can lead to important deviations, especially for deep-seated tumors, potentially resulting in a suboptimal approach with incomplete exposure. Augmented reality (AR) allows displaying of the tumor and critical structures directly on the patient, which can simplify and improve surgical preparation.

**Methods:**

We developed an AR-based workflow for intracranial tumor resection planning deployed on the Microsoft HoloLens II, which exploits the built-in infrared-camera for tracking the patient. We initially performed a phantom study to assess the accuracy of the registration and tracking. Following this, we evaluated the AR-based planning step in a prospective clinical study for patients undergoing resection of a brain tumor. This planning step was performed by 12 surgeons and trainees with varying degrees of experience. After patient registration, tumor outlines were marked on the patient's skin by different investigators, consecutively using a conventional neuronavigation system and an AR-based system. Their performance in both registration and delineation was measured in terms of accuracy and duration and compared.

**Results:**

During phantom testing, registration errors remained below 2.0 mm and 2.0° for both AR-based navigation and conventional neuronavigation, with no significant difference between both systems. In the prospective clinical trial, 20 patients underwent tumor resection planning. Registration accuracy was independent of user experience for both AR-based navigation and the commercial neuronavigation system. AR-guided tumor delineation was deemed superior in 65% of cases, equally good in 30% of cases, and inferior in 5% of cases when compared to the conventional navigation system. The overall planning time (AR = 119 ± 44 s, conventional = 187 ± 56 s) was significantly reduced through the adoption of the AR workflow (*p* < 0.001), with an average time reduction of 39%.

**Conclusion:**

By providing a more intuitive visualization of relevant data to the surgeon, AR navigation provides an accurate method for tumor resection planning that is quicker and more intuitive than conventional neuronavigation. Further research should focus on intraoperative implementations.

## 1. Background

Prior to the resection of intracranial tumors, careful planning and preparation are required. This entails the review of preoperative medical imaging data as well as the preparation of the computer-assisted surgical navigation hardware (e.g., neuronavigation system), allowing the surgeon to plan an optimal approach to the tumor. The plan consists of an entry point and a target; the entry point defines the center of approach around which the craniotomy will be made, the target being the whole tumor. Often, this plan is refined immediately prior to the intervention, after patient registration, when the tumor outline is drawn on the patient's skin to serve as a guide for incision and craniotomy.

**Figure 1:**
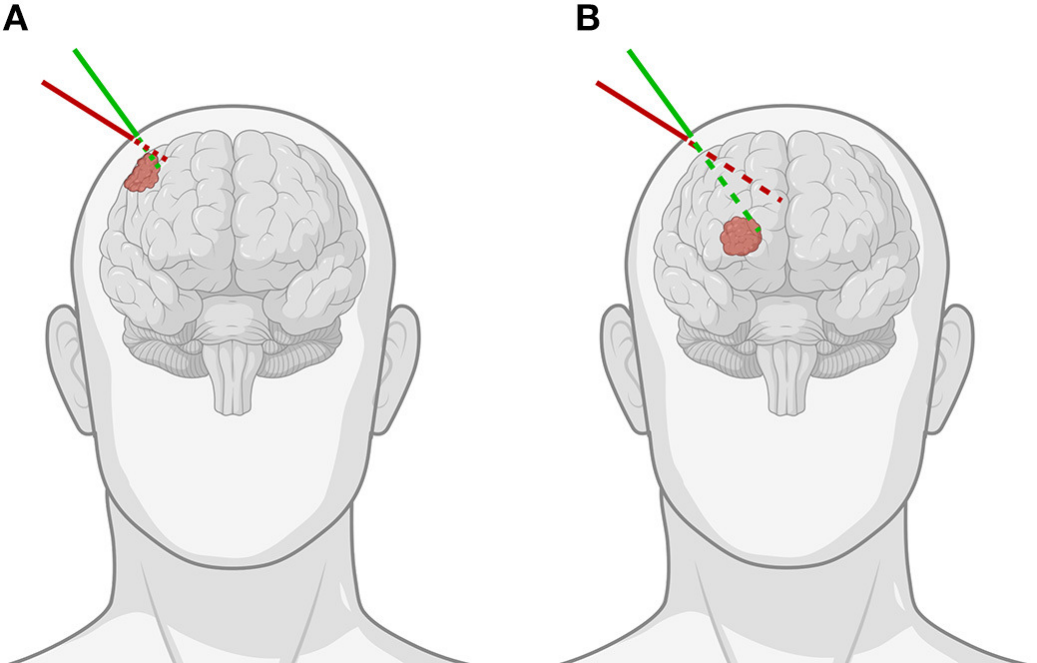
Demonstration of the importance of the angle of approach when using pointer-based neuronavigation for **(A)** superficial as opposed to **(B)** deep-seated tumors. The green line indicates the correct angulation of the stylus for the chosen entry point, while the red line shows how for the same entry point, improper angulation can lead to important differences in trajectory and offset to the tumor, especially for deep-seated tumors [Created with BioRender.com].

A neuronavigation system is often used to define a virtual three-dimensional (3D) coordinate system around a fixed patient and spatially align any prior medical imaging data to the patient within that coordinate system. This process is known as registration and can be achieved through accurate tracking of a hand-held stylus within the virtual coordinate system. The stylus allows the surgeon to identify points on the patient's face or localize fiducials. These data serve as the target for aligning imaging data. Following this, the tracked stylus is then used to localize tumor borders, which serve as a basis for the plan. The drawbacks of this approach are inherent limitations of current state-of-the-art neuronavigation systems. First, visualization of the imaging data used to guide the surgeon is typically carried out on secondary displays, resulting in a disconnect between the surgeon and the patient. Second, the 3D imaging data are often represented as two-dimensional (2D) projections, either orthogonal multiplanar reconstructions or oblique views in line with the stylus. This implies that the user performs a mental transformation between the 2D space on the monitor and the 3D space of the patient, often without the ability to focus on both spaces concurrently. Although 3D visualization techniques such as volume rendering or segmented surface models aid in the interpretation of the imaging data, the disconnection in space and the cognitive burden of the transformation still remain (1). The consequence of these limitations is that tumor margins may be underestimated or overestimated, or that the angle of approach may diverge from the planning, a problem exacerbated in deep-seated tumors, potentially resulting in a suboptimal approach with incomplete exposure (Figure 1).

Augmented reality (AR) has the potential to address these limitations, by providing a means to view relevant 3D medical imaging data superimposed onto the patient. This provides a more intuitive data representation as it keeps the surgeon's focus on the patient and reduces the mental load of transforming information (1, 2). Ivan et al. (3) previously proposed such a solution for AR-assisted tumor delineation using the HoloLens II (3). In their small pilot study, the commercially available OpenSight software was used to display volume-rendered patient data which had been manually registered with the patient's anatomy. The reported results were promising and hinted toward feasibility, although no direct tracking of the patient nor a quantifiable registration method was implemented. Therefore, it is impossible to conclude the true accuracy of such an AR workflow based on this study alone.

**Figure 2:**
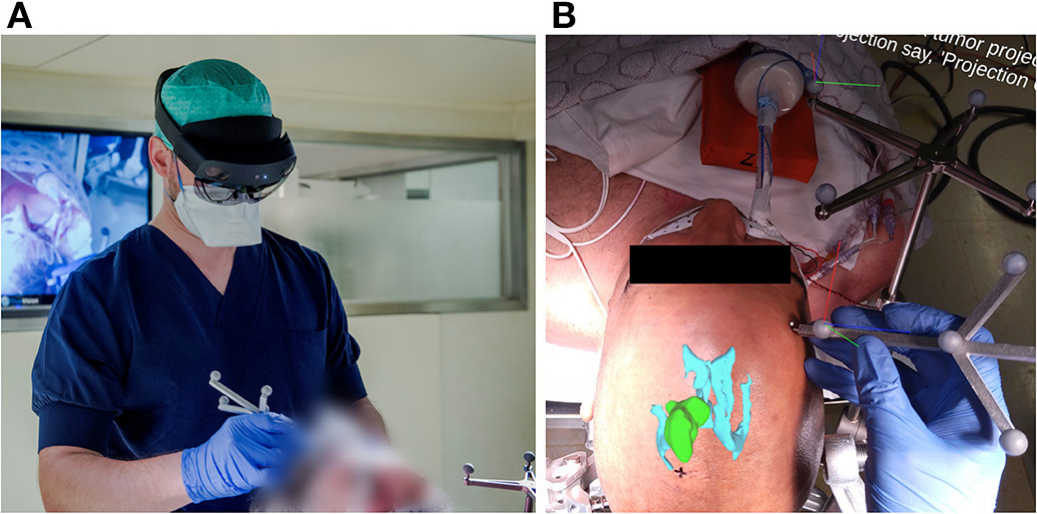
**(A)** Investigator wearing the AR headset and performing the planning step with the AR application for guidance, using an in-house designed handheld stylus and a stationary reference star, adopted from the Brainlab Curve neuronavigation system. **(B)** View from within the AR headset, as seen by the investigator at the moment after patient registration and before tumor projection and delineation. The AR overlay shows the 3D tumor and ventricle models in their correct anatomical position. The black cross marked on the patient's skin indicates the central entry point, as predefined by the experienced neurosurgeon. The RGB axes visible on the Brainlab reference star indicate the established 3D cartesian coordinate system in which the patient's position is being tracked. The RGB axes visible on the handheld stylus indicate its correct tracking, along with the white dot aligned on its tooltip.

To address the current limitations of conventional neuronavigation systems and off-the-shelf AR hardware, we developed an AR-based navigation system deployed on the Microsoft HoloLens II (Figure 2), which exploits the built-in depth-camera for infrared tracking. This allows for more robust tracking compared to RGB-camera tracking methods and also has the practical advantage that it can depend on the infrared-reflecting spheres and constellations thereof that are already widely used by conventional neuronavigation systems (4–6). The proposed method, thus, focuses on providing the familiar workflow and comparable performance of existing commercial neuronavigation solutions through a high-accuracy tracking algorithm, but without requiring external tracking cameras, ancillary displays, or a separate computer system. We aimed to validate the AR workflow for tumor resection planning in terms of accuracy and duration, in a direct comparison with a current state-of-the-art neuronavigation system. First, we performed phantom testing to ensure that the AR system's registration accuracy was on par with the conventional system. Following this, we tested the AR system in a clinical trial for patients undergoing brain tumor resection.

## 2. Materials and methods

### 2.1. Pre-clinical registration evaluation

**Figure 3:**
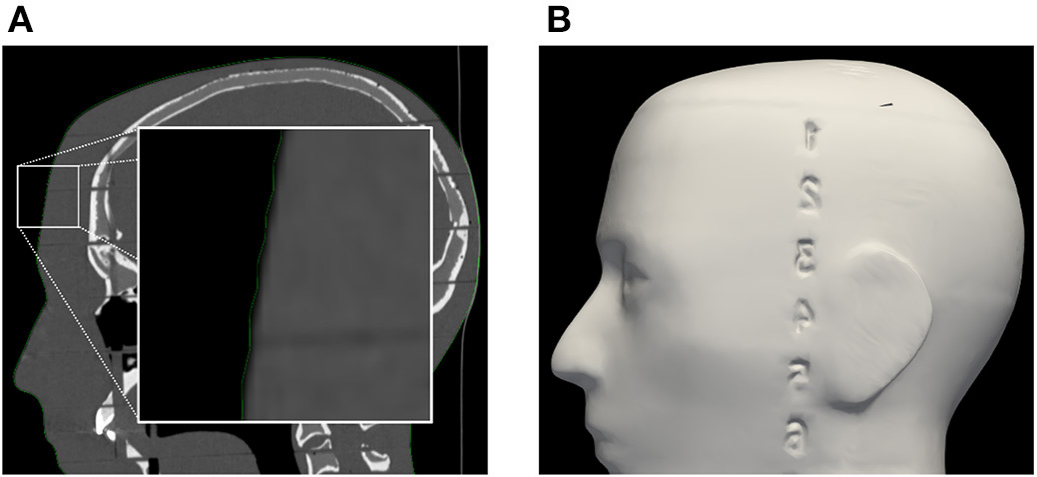
**(A)** Segmentation of phantom CT data used for the creation of **(B)** the target surface-model during pre-clinical registration validation. Note: the enlarged region in the left image indicates a minor surface bias ( ≤ 1 mm) in all dimensions of the surface model (green outline). This bias was discovered in retrospective analysis and could be due to thresholding and partial volume effect of the CT scan images.

**Figure 4:**
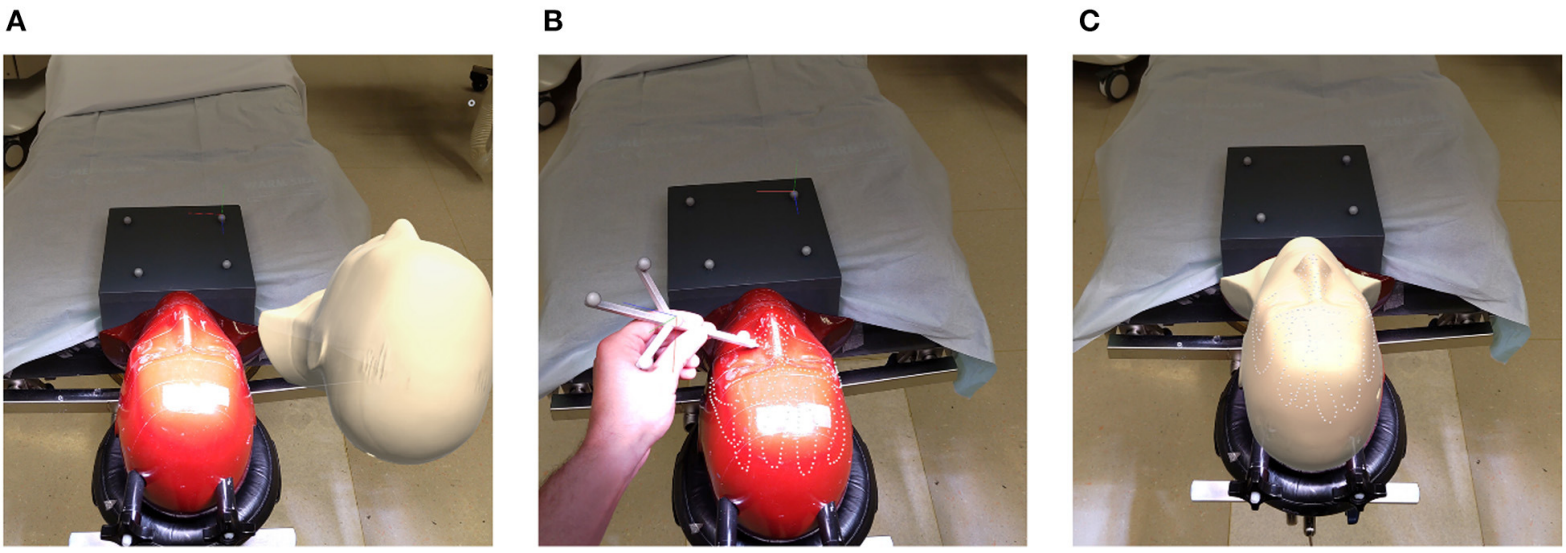
Registration pipeline: **(A)** unregistered phantom; **(B)** refining initial alignment; **(C)** registered phantom.

Registration performance of the AR-based navigation system was evaluated through a phantom study. A phantom consisting of an Alderson Radiation Therapy Phantom (slices 1–9) (Radiology Support Devices Inc., Long Beach, California, USA) rigidly affixed to a reference star with four infrared-reflecting spheres (Northern Digital Incorporated, Waterloo, Canada) was CT scanned (Figure 3). This scan was preprocessed using the 3DSlicer image processing software (http://slicer.org, version 4.10.2) to produce a 3D “skin” model of the phantom head (7). The 3D coordinates of the passive infrared-reflecting spheres in CT space were logged and used to define the reference star pose for the application to track, effectively pre-registering the phantom model. Any error in subsequent registration would manifest as a deviation from this identity registration. In total, 20 phantom registrations were performed in an operating room setting. Ten registrations were carried out using an AR workflow, deployed on the HoloLens II (Microsoft Corporation, Redmond, Washington, USA) (Figure 4), while 10 were carried out using a conventional neuronavigation system (Brainlab Curve 2; Brainlab AG, Munich, Germany). The registration transform from the conventional system was logged to a local PC by means of the OpenIGTLink API (http://openigtlink.org). A ground truth registration transform was defined through least-square-fit between the infrared-reflecting sphere coordinates in CT space and their corresponding coordinates in local tracker space. The latter was established through caliper measurements (Mitutoyo 530–112). The registration quality was calculated as the relative transformation to the ground truth.

### 2.2. Prospective study design and patient inclusion

A prospective clinical pilot study was designed to evaluate registration and tracking accuracy as well as the practical advantages of the developed AR navigation system. The primary endpoint was the validation of the registration and delineation accuracy in a direct comparison with a conventional neuronavigation system, and the secondary endpoint was the time it took to perform the preoperative surgical planning. Adult patients at the University Hospital of Brussels (Universitair Ziekenhuis Brussel, Vrije Universiteit Brussel) diagnosed with an intracerebral tumor and planned for resection surgery were eligible for inclusion in the study. Exclusion criteria included patients with extra-axial tumors and those requiring prone positioning during the intervention. The study protocol was approved by the Ethics Committee of UZ Brussel and validated by the Belgian Federal Agency of Medicines and Health Products (FAGG/AFMPS). All patients provided written informed consent.

### 2.3. Resection planning protocol

A total of 12 surgeons and trainees with varying degrees of experience were involved in this study. Resection planning was performed preoperatively with the patient positioned on the operating table, the head rigidly fixed in a Mayfield clamp. At the start of the experiment, the expert neurosurgeon performing the surgery defined the angle of approach for the surgery by marking the central entry point. This point served as the reference for tumor delineation for both the conventional neuronavigation and the AR system. During the experiment, the planning was performed in two steps: first the registration step by using a tracing method with a tracked pointer, and second the delineation step indicating the tumor outlines, projected on the patient's skin from the chosen approach angle, with a marker. The registration step and delineation step were both timed. After registration, the person performing the tasks could gather the necessary equipment and proceed to the delineation step without being timed.

**Figure 5:**
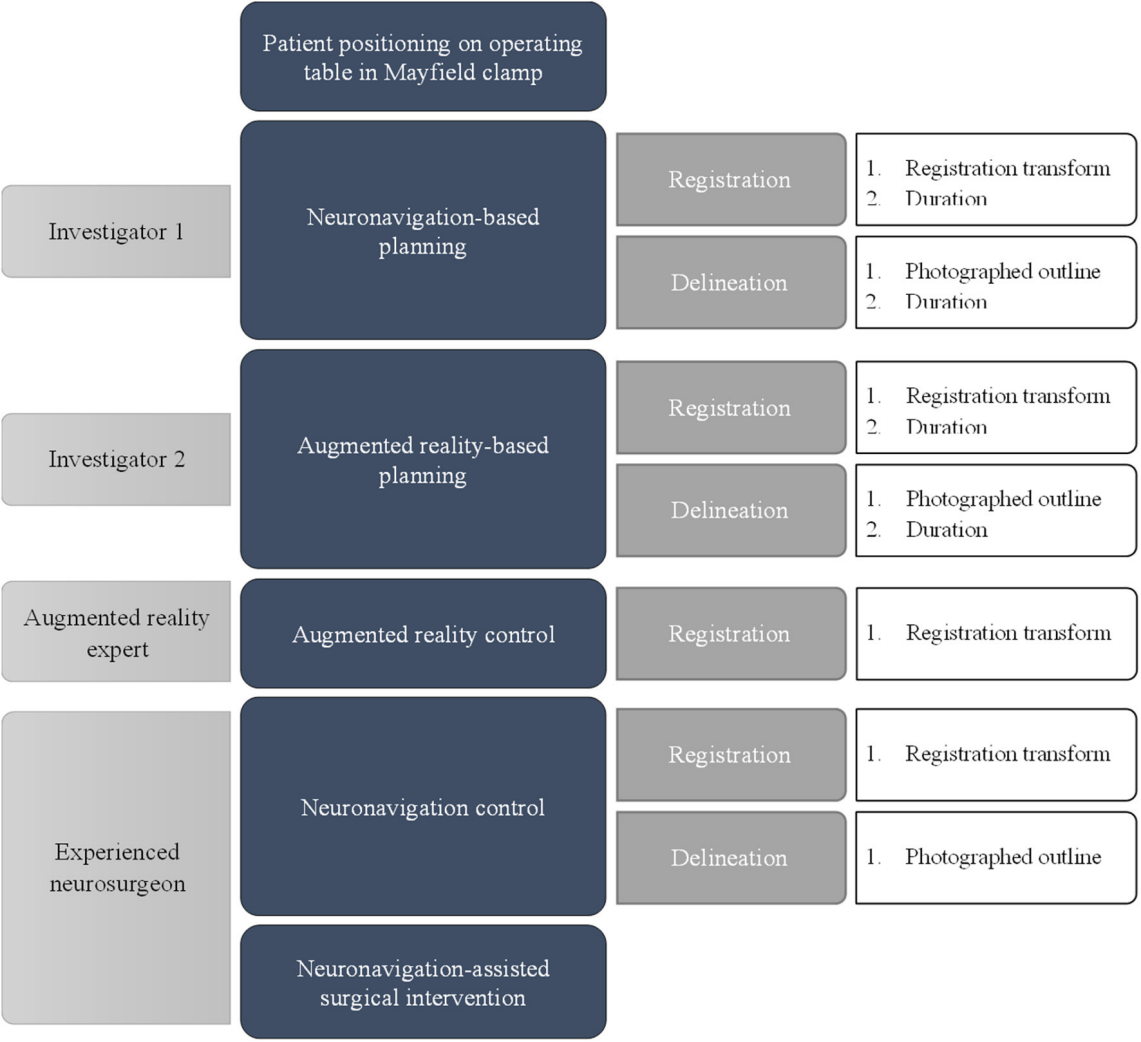
Flow diagram of the different experimental phases for a single case.

A flow diagram of the experiment for a single case is shown in Figure 5. The planning procedure of a single case was executed by a first investigator using a conventional neuronavigation system, followed by a second investigator using the proposed method and an AR head-mounted device (HMD). After logging each performance, the tumor delineation was erased from the skin. Afterward, the AR-based registration step was performed by an AR expert to log the registration transform data from an experienced user. Then, the complete planning was performed by an experienced neurosurgeon, and both the registration transform and tumor delineation using the conventional navigation system were recorded. This was considered the gold standard for accuracy to which we compared the AR and conventional navigation-based planning performed by the investigators. Then the standard surgical procedure was performed by an experienced neurosurgeon using information from the conventional neuronavigation system.

### 2.4. Guidance techniques

#### 2.4.1. Conventional neuronavigation

Conventional navigation was performed using the Brainlab Curve system, and the included infrared tracked stylus was used to register preoperative medical imaging data to the fixed patient. Following the device protocol, we sequentially identified three patient landmarks (left/right lateral canthus, nasion) using the stylus to define an initial alignment. Next, a sparse point cloud was sampled from the patient's skin, which the system used to refine the registration. Following this, the investigator verified the registration by identifying recognizable landmarks on the patient's skin and checking for correspondences on the navigation system.

Upon completion of the registration, the investigator proceeded to the tumor delineation. From the predefined central entry point, the investigator identified the anterior, posterior, medial, and lateral borders of the tumor using the tracked pointer and inspecting the inline views of the imaging data on the external screen and outlined these on the patient's skin. When the investigator was satisfied with the result, completion was confirmed.

#### 2.4.2. Augmented reality

##### 2.4.2.1. Data preprocessing

Preoperatively, surface meshes were generated based on the patient's preoperative medical imaging data using Brainlab Elements software. This resulted in a skin, cerebrum, and tumor model. The surface meshes were post-processed to reduce vertex count using quadric-edge-collapse decimation, with a target vertex count of approximately 10,000 each. In addition, we logged four 3D landmarks defining the patient's left/right lateral canthus, nasion, and tip-of-nose. These models and 3D landmark files were loaded into the AR application prior to the procedure.

##### 2.4.2.2. Application

The application to be deployed on the HoloLens II AR-HMD was developed using the Unity game development environment (version 2019.4.28f1) and incorporated inside-out infrared tracking (8). A handheld stylus and a stationary reference star were used for navigation (Figure 2). The stylus was designed and manufactured in-house, machined from 6061 aluminum 5 mm stock using a Modela Pro II MDX-540 3D Milling Machine (Roland DG Corporation, Hamamatsu, Japan), and supported a constellation of five infrared-reflecting spheres. The reference star was adopted from the Brainlab Curve neuronavigation system, allowing a one-to-one mapping between the AR and Brainlab coordinate systems.

To be able to compare results between techniques but also to minimize any learning curve, the workflow of the application was designed to mimic the workflow of the neuronavigation system and consisted of three key stages. The first is the definition of a 3D coordinate system around the patient. This is performed by defining a coordinate system around the Brainlab reference star, whose tracked pose served as a basis for all data processing. The application advances to the next stage only after establishing a stable star-defined coordinate system.

**Figure 6:**
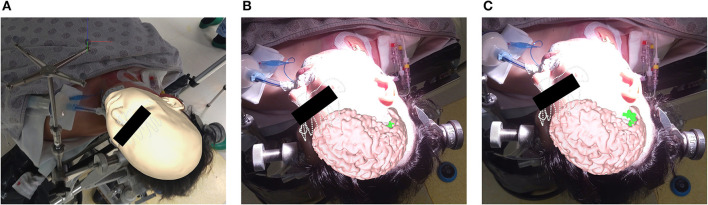
Example of the sequence that the investigators followed within the AR application. **(A)** Patient installed in the Mayfield clamp, with the Brainlab reference star rigidly attached. The RGB axes visible on the Brainlab reference star indicate the established 3D cartesian coordinate system in which the patient's position is being tracked. The virtual skin model is matched to the patient's anatomy during the registration step, in which the boney surfaces along the patient's face are digitized with the handheld stylus, resulting in a point cloud (white dots). **(B)** After registration, the virtual patient anatomy is displayed in its actual location. Here the cerebrum is shown in pink, with a deep-seated tumor recurrence (located in a previous resection cavity) shown in green. **(C)** An orthographic projection of the tumor is shown on the patient's skin, allowing delineation of the tumor contours.

The next stage entails the registration between medical imaging data and the patient (Figure 6). Using the tracked stylus, the surgeon identifies the left and right lateral canthus, the nasion, and the tip-of-nose of the patient. Based on least-square-fitting, these landmarks are registered to their corresponding points predefined in the model. As such, an initial crude registration between the model and the patient is initiated. Using the hand-held stylus, the surgeon then captures additional 3D points from the boney surfaces along the patient's face. Based on these points and an iterative closest point algorithm, the registration is refined. After each registration step, the mean point-to-point error metric is displayed to the surgeon, allowing him/her to refine the final registration, if need be, by replacing and/or collecting more points. Registration is finalized after visual inspection and vocal confirmation by the surgeon.

**Figure 7:**
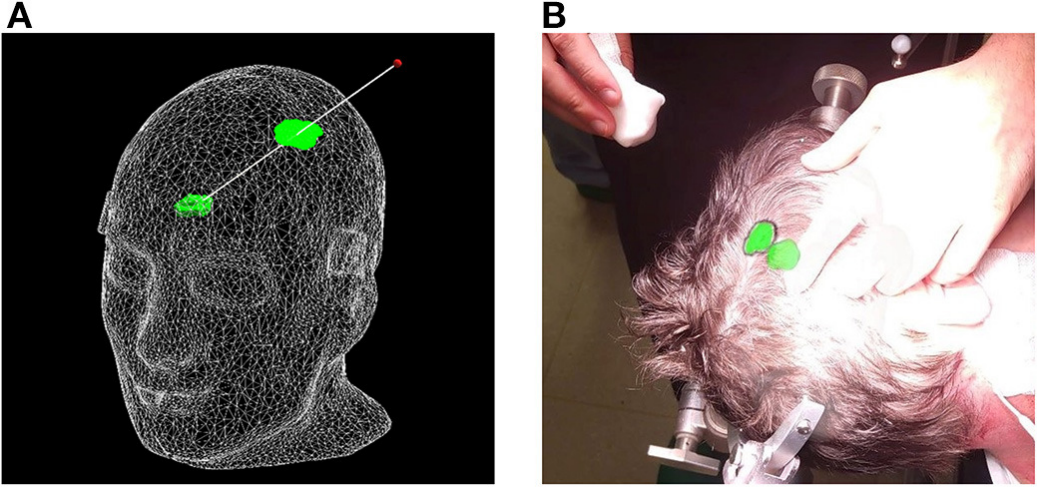
Proposed surface-based tumor projection. **(A)** Wireframe model of the phantom with a deep-seeded tumor (green) projected upon the skin (green outline) nearest to the handheld stylus' tip (red dot). The trajectory between the tumor and stylus is illustrated by the white line, and along with the wireframe skin, it is shown here purely for illustrative purposes and is not visible in the AR application. **(B)** Example of a clinical case, with the tumor displayed in its actual position as a 3D model inside the patient's head, along with the orthographic projection on the patient's skin, allowing the delineation of the tumor (black marker).

The third and final stage is planning. For this, we display an orthographic projection of the tumor (originating from its center of mass) which follows the stylus' tip position (Figure 7). The tumor projection is determined from the intersection of the skin surface mesh and the orthographic projection of the tumor surface mesh along the vector between the tumor's center of mass and the stylus' tip position. As such, the tumor is projected onto the irregular surface of the registered skin model. As the surface model of the skin is made transparent, the projection of the tumor appears to follow the contour of the patient's skin. Using the stylus, the tumor projection is placed by the surgeon at the predefined central entry point and fixed in place through a verbal command. Since it is an orthographic projection to this point, it is automatically centered on the axis through the entry point and the center of mass of the tumor. Therefore, it is not essential to keep the stylus parallel to this axis, as is the case for the conventional neuronavigation system. The planning step is finalized by tracing the contour of the projection to the patient's skin using a marker.

### 2.5. Clinical outcomes

For each clinical case, the planning using either guidance technique was performed by two different investigators in order to attenuate learning effects resulting from the first technique. Following the above protocols, both trainee and expert surgeons performed clinical trials using both devices. This provided insight into operator variance for each system at varying skill levels. To average out interindividual differences in planning accuracy and duration, the investigators were rotated over both guidance techniques. Tumor delineation accuracy was evaluated by direct comparison between the delineation resulting from either the AR or the neuronavigation workflow and a delineation of the expert surgeon using neuronavigation. For this purpose, photographs from a fixed camera viewpoint were used. The investigator delineation was assessed by the expert neurosurgeon and scored in terms of resemblance to the expert delineation and detail of delineation. Workflow duration, including registration and delineation steps, for both the neuronavigation system and AR system, was timed using a stopwatch.

### 2.6. Statistical analysis

Statistical analysis was performed in Python (3.9) with the SciPy statistics library (1.7.0). Comparison between workflow duration data for both the AR and conventional techniques was assessed through Welch's *t*-test. The comparison of registration variance between both techniques was assessed through a one-way f-test. We considered *p*-values of < 0.05 to be statistically significant.

## 3. Results

### 3.1. Pre-clinical registration evaluation

**Figure 8:**
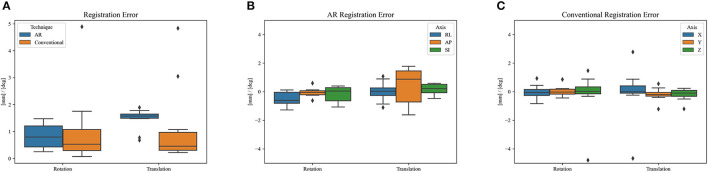
Phantom registration error of AR and conventional neuronavigation techniques shown as an error in rotation and translation. **(A)** Total registration error magnitudes; **(B)** AR registration error vectors (RL, right-left; AP, anterior-posterior; SI, superior-inferior; orientations defined in CT space). **(C)** Conventional neuronavigation registration error vectors. The black diamonds represent outlier data.

**Figure 9:**
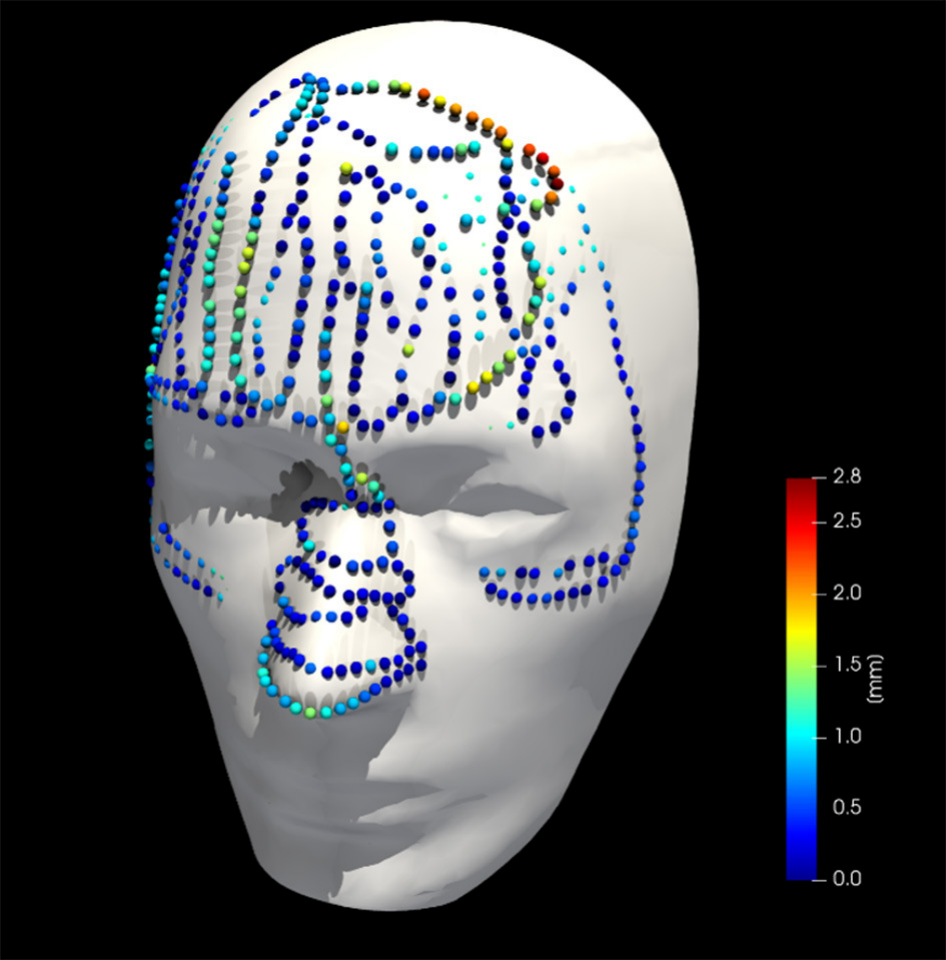
Collected point cloud data and registered phantom for a single case; point colors are scaled to point-to-surface distance.

Figure 8 shows the phantom registration error, displayed as an error in translation and rotation, for both AR navigation and conventional neuronavigation. During phantom experiments, the AR device demonstrated a mean registration error of 1.42 ± 0.42 mm and 0.95 ± 0.36° (Figure 8). This was not statistically different (*p* = 0.57) from the conventional navigation system: 1.17 ± 1.46 mm and 1.05 ± 1.37°. However, with outlier data removed (median absolute deviation, cutoff of 2.5) from conventional neuronavigation registration data, the registration error was reduced to 0.39 ± 0.14 mm and 0.38 ± 0.19°, which was statistically better than the registration performed on the AR device (*p* = 0.014). The root mean square point-to-plane distances (Figure 9) between the point cloud and registered phantom overall registration data was 0.70 ± 0.12 mm using the AR device.

### 3.2. Clinical assessment

**Figure 10:**
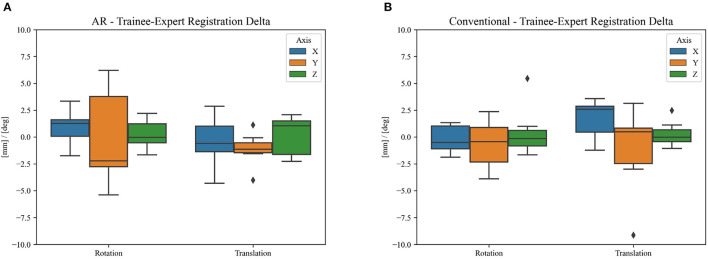
Difference in clinical registration between expert and trainee investigators using **(A)** the proposed AR workflow and **(B)** the conventional neuronavigation workflow. The registration variability between expert and non-expert surgeons using the proposed AR method was not statistically different (*p* = 0.14) when compared to the traditional neuronavigation system. The black diamonds represent outlier data.

**Figure 11:**
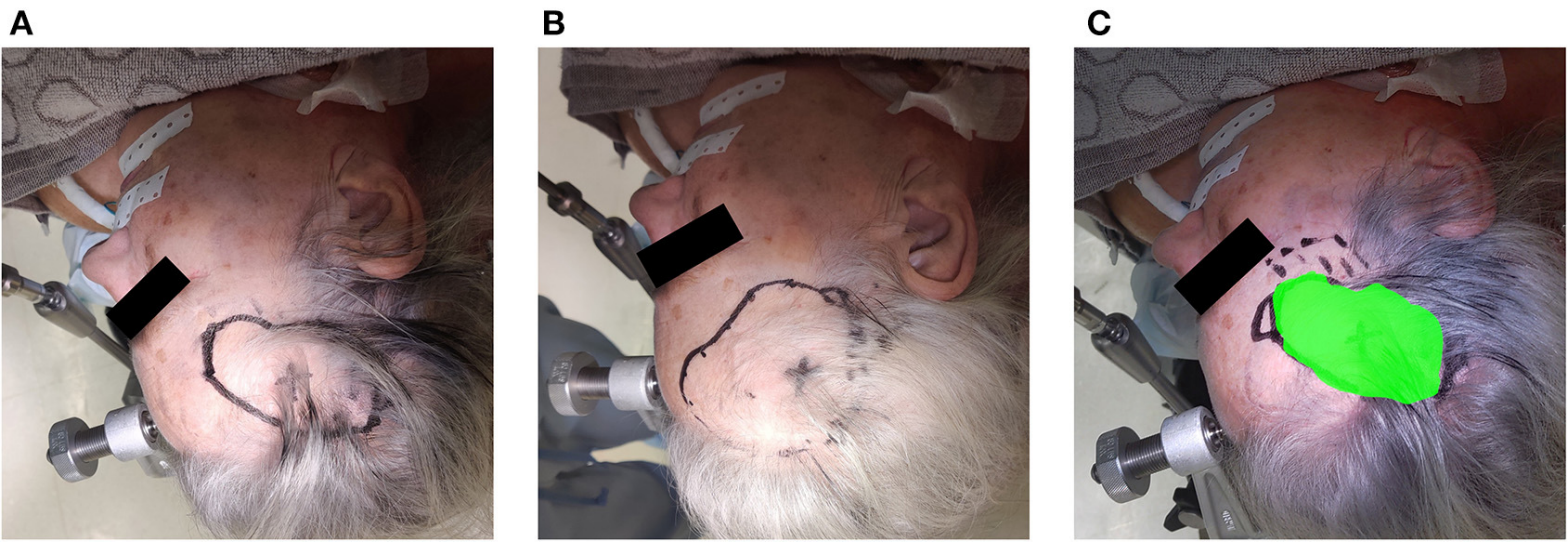
Example of the assessment of delineation accuracy: **(A)** delineation using the AR system; **(B)** delineation using the conventional neuronavigation system; **(C)** control based on expert delineation.

Over a 6-month period, 20 patients were included with a mean age of 61 years [35–76], 11 were men and 9 were women. All patients presented with one or more intracerebral tumors planned for resection. Of these tumors, 9 were metastases, 6 were recurring glioblastomas, and 5 were newly diagnosed glioblastomas. This included 12 frontal, 6 temporal, and 2 parietal tumors; 16 right-sided and 4 left-sided; and 17 superficially located and 3 deep-seated. Registration variability between non-expert and expert surgeons using the AR-HMD was not statistically different (*p* = 0.14) when compared to the traditional navigation system (Figure 10). With respect to qualitative assessment of tumor delineation accuracy, AR-guided planning was deemed superior in 65% of cases (*n* = 13), equally good in 30% of cases (*n* = 6), and inferior in 5% of cases (*n* = 1) when compared to the conventional navigation system (Figure 11). The delineations were generally more detailed, displaying more intricate outlines, in comparison to the typical circle or rectangle that is drawn with the conventional neuronavigation system. Because of the incorrect interpretation of the approach angle and inline views (Figure 1), the conventional neuronavigation workflow also tended to result in an overestimated tumor size (Figure 11).

**Figure 12:**
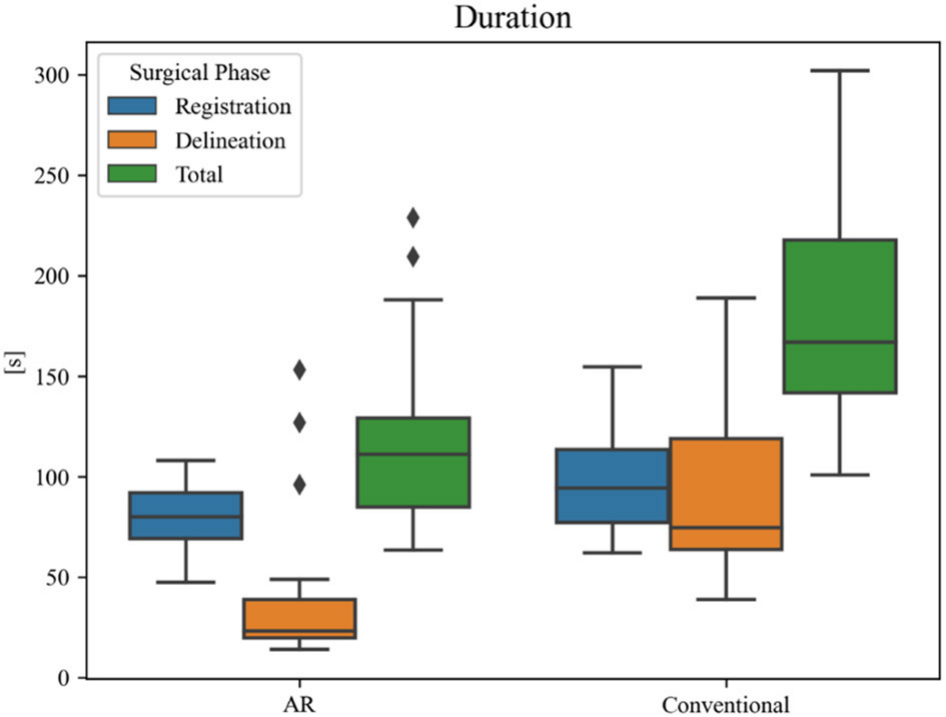
Duration of surgical planning phases for each technique. The black diamonds represent outlier data.

Overall planning time (AR = 119 ± 44 s, conventional = 187 ± 56 s) was significantly reduced when using AR navigation (*p* < 0.001) (Figure 12). In 90% (18 out of 20) of the cases, subjects displayed a superior working speed with an average time reduction of 39%, which is equivalent to 1 min 17 s. This is mostly due to the reduction in tumor delineation time (*p* < 0.001), though a significant reduction in registration time between the techniques was also seen (*p* = 0.014). Registration and tumor delineation using AR navigation was subjectively assessed to be more intuitive by all test subjects.

## 4. Discussion

We performed a prospective clinical pilot study investigating the accuracy and efficacy of an AR solution toward tumor resection planning. Our primary goal was to demonstrate that the registration and delineation accuracy of the AR approach met the accuracy performance of a conventional neuronavigation system. The evaluation on a phantom head indicated that the registration workflow of the AR system was accurate and could perform adequately in an operating room environment. During our clinical trial in the operating room, we demonstrated that AR assistance was quick and intuitive, and allowed accurate tumor resection planning in a neurosurgical setting. The proposed system closely matched the registration quality of the state-of-the-art neuronavigation system, while improving the accuracy and reducing the duration of the resection planning.

**Figure 13:**
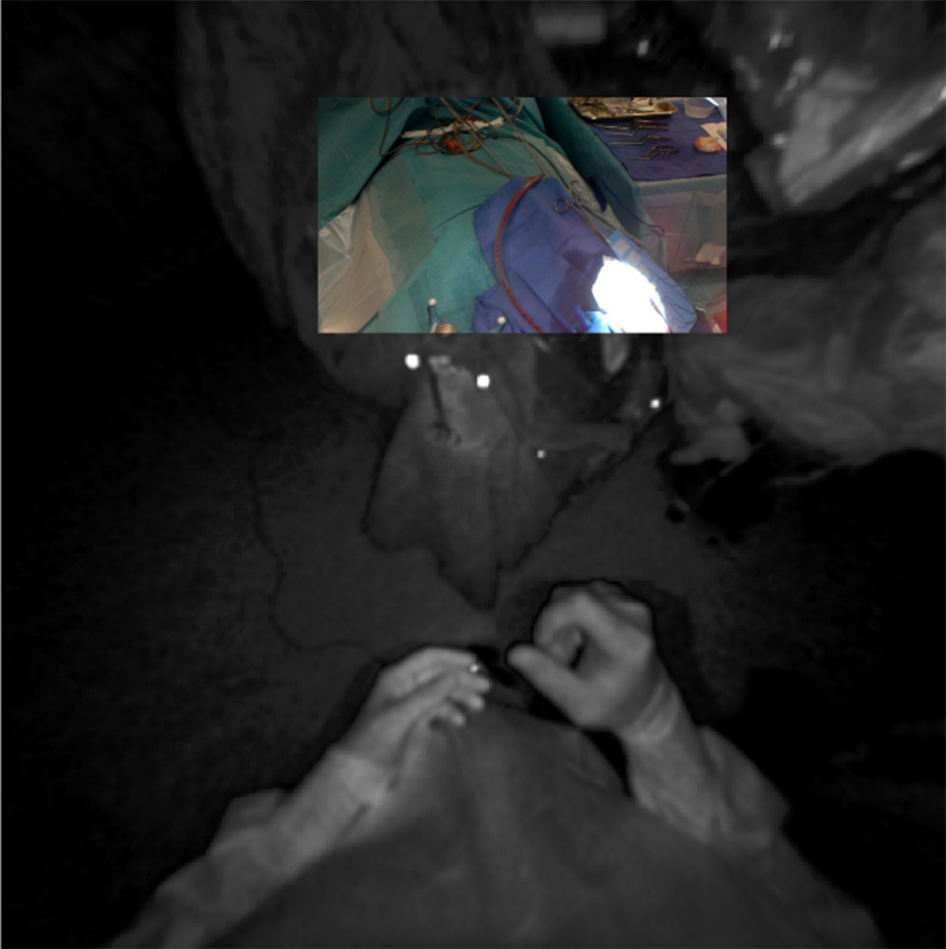
Registration of RGB and infrared sensor maps on the HoloLens device, demonstrating the more favorable tracking frustum afforded by the infrared sensor's downturned orientation and wider field-of-view (180°).

Other research groups have focused on the use of off-the-shelf AR-HMDs for surgical navigation, as these devices often contain much of the hardware required for computer-aided navigation, i.e., computational power, display, and tracking. However, since the current consumer-grade HMDs have a tracking error of approximately 2 cm and offer no direct tracking of the patient, nor a quantifiable registration method, a dedicated tracking optimization needs to be developed before surgical use is possible (9). Various approaches have been attempted to obtain more accurate tracking. Manual alignment of AR visualizations based on visual interpretation was shown to be unreliable and inconsistent, with a variance in target registration error (TRE) of up to 278% (4). Tracking of QR code-style markers using the RGB camera, as described by Fick et al., on the contrary, resulted in a mean fiducial registration error of 8.5 mm (10). This method also has several practical limitations, such as the large size of the markers, the small field-of-view of the RGB sensor (48° for image processing tasks), and the orientation of the RGB sensor which is ill-suited for surgery due to a less than ideal trackable frustum at working distances (Figure 13) (4, 6). Gsaxner et al. proposed tracking based on real-time face detection and dense point cloud sampling from the time-of-flight depth sensor and reported a TRE of 9.2 ± 1.5 mm (11). There was no compensation for tracking drift over time in their study, however, requiring the face to be continuously visible, which is impractical in a surgical setting. Inside-out infrared tracking using the HoloLens' on-board depth and infrared sensor resolves many of these practical issues. The tracking of infrared-reflecting spheres, which are routinely utilized in current neuronavigation, provides a precise static coordinate system and digitization mechanism (5). Moreover, the trackable frustum is significantly increased (Figure 13). For these reasons, we developed an infrared tracking algorithm that allows AR visualizations with less perceived drift and high accuracy (0.78 ± 0.74 mm; Frantz et al., unpublished results) (6, 8, 12). This tracking algorithm facilitated the successful implementation and promising results described here.

In terms of registration accuracy, the calculated ground truth registration resulted in a mean point-to-point transformation error of 0.39 mm between the estimated local infrared-reflecting sphere coordinates of the Brainlab star and their corresponding position in CT space, indicating an acceptable ground truth to compare against. The registration performance obtained on the AR device (1.42 ± 0.42 mm and 0.95 ± 0.36°) surpasses prior state-of-the-art AR-HMD registration quality of 3.9 ± 1.8 mm and 4.9 ± 2.4° and is nearing parity with commercial systems (11). The presence of several outlier data in the conventional neuronavigation registration results highlights that these purpose-built systems are not failproof, even when operated by expert users. The non-significant variability between non-expert and expert surgeons with respect to registration suggests that the AR registration workflow, which was modeled on the neuronavigation system, resulted in a similar learning curve, although it was significantly faster. We discovered a slight inflation of the generated phantom surface mesh (Figure 6). This surface bias is one possible explanation for the AP error observed in the AR registration results (Figure 8).

In 95% of cases, the AR workflow produced tumor delineations that were equally good or better, and generally more detailed in comparison to the conventional neuronavigation system. Apart from the more intuitive visualization, this can be explained by the orthographic projection of the tumor on the patient's skin. Since it no longer required the surgeon to keep the stylus parallel to the central angle of approach to obtain an accurate projection of the tumor on the skin surface, there were fewer errors resulting from incorrect angulation of the stylus when using the AR workflow. As such, AR-based planning, in combination with a high-accuracy tracking and registration method, might be more accurate. This further expands on the results obtained by Ivan et al., already showing promising results in terms of tumor delineation but lacking accuracy in the AR system (3). In addition, the proposed AR workflow was significantly faster than the conventional neuronavigation for the entire resection planning (*p* < 0.001) (Figure 12), with an average time reduction of 39%. This was mostly due to the reduction in tumor delineation time (*p* < 0.001), highlighting the increased intuitiveness for tumor delineation using the AR visualization. This intuitiveness is corroborated by the significant reduction in registration time (*p* = 0.014) in the absence of a significant difference in registration variability compared to the conventional navigation system. The abovementioned results, in addition to our previous findings on intracranial drain placement, lead us to hypothesize that AR has the ability to flatten the learning curve and increase surgeon confidence for tumor resection planning as well, by making the planning step more visual in nature (12). However, we did not assess individual performance over time to corroborate this hypothesis.

From a technical standpoint, there were two prominent limitations. The first was the lack of a known ground-truth local geometry of the Brainlab reference star, necessitating an estimation. This limited the quality of a ground truth registration transform when quantifying the performance of the Brainlab system. Second, the handheld stylus used in the proposed AR solution was pre-calibrated, and the tooltip location was taken as a constant throughout the trial. It is possible that this could have been corrupted through mechanical means, resulting in inaccurate data sampling. This could also explain the AP registration error observed during phantom trials (Figure 8). With respect to data analysis, there were two more limitations. The first was the qualitative assessment of the tumor delineation accuracy by an expert neurosurgeon. A quantitative comparison of the delineations from each guidance technique, such as the one proposed by Ivan et al., could have mapped the correspondence between both techniques more objectively (3). Nevertheless, the validation by the expert neurosurgeon, combined with the objectively confirmed registration accuracy, already provides an important insight into the improved accuracy and detail obtained with the AR guidance. Second, apart from the division between experts and non-experts, no further distinction was made between the different levels of surgical training of the investigators. This makes it impossible to ascertain any source of errors within the group, as it might result from a lack of experience in using conventional neuronavigation. However, the group of investigators was sufficiently diverse in terms of experience to make general assumptions about the ease-of-use of the proposed AR method.

Further research should focus on expanding and evaluating the use of high-accuracy AR-HMD guidance intraoperatively, where experienced neurosurgeons use the AR system not only during the planning step but also throughout later steps of the surgery while still using the conventional neuronavigation system as the principal source of surgical guidance. Since magnification is often not possible on these HMDs, the implementation of an exoscope as a source of magnification, in combination with an AR or VR headset, should be explored and compared to an AR infusion already available in the microscope.

### 4.1. Conclusion

We developed an AR navigation system, deployed on the HoloLens II AR-HMD, that matches the registration accuracy of a state-of-the-art neuronavigation system. By providing a more intuitive visualization of relevant data to the surgeon, the AR navigation workflow provides an accurate method for tumor resection planning that resulted in more detailed tumor delineations and reduced preoperative planning time when compared to the conventional neuronavigation system. Further research should focus on intraoperative implementations.

## Data availability statement

The raw data supporting the conclusions of this article will be made available by the authors, without undue reservation.

## Ethics statement

The studies involving human participants were reviewed and approved by the Ethics Committee of UZ Brussel and validated by the Belgian Federal Agency of Medicines and Health Products (FAGG/AFMPS). The patients/participants provided their written informed consent to participate in this study. Written informed consent was obtained from the individual(s) for the publication of any potentially identifiable images or data included in this article.

## Author contributions

FV, TF, WG, MB, BJ, TS, JV, and JD: research design. FV, TF, FB, WG, QN, MB, and JD: acquisition of data. FV, TF, FB, JV, and JD: analysis and interpretation of data. FV, TF, FB, WG, TS, JV, and JD: drafted the manuscript. All authors: critically revised the manuscript. All listed authors have made substantial contributions to the presented study, read, and approved the final submitted manuscript.
